# The analgesic action of larixyl acetate, a potent TRPC6 inhibitor, in rat neuropathic pain model induced by spared nerve injury

**DOI:** 10.1186/s12974-020-01767-8

**Published:** 2020-04-16

**Authors:** Jing Wang, Ming Zhao, Peng Jia, Fang-Fang Liu, Kun Chen, Fei-Yang Meng, Jiang-Hao Hong, Ting Zhang, Xiao-Hang Jin, Juan Shi

**Affiliations:** 1 Department of Human Anatomy, Histology and Embryology & K. K. Leung Brain Research Centre, Preclinical School of Medicine, The Fourth Military Medical University, Xi’an, 710032 China; 2 Student Brigade, Preclinical School of Medicine, The Fourth Military Medical University, Xi’an, 710032 China; 3 Department of Neurobiology, Preclinical School of Medicine, The Fourth Military Medical University, Xi’an, 710032 China; 4 Department of Basic Medical Morphology, Medical College, Xijing University, Xi’ an, 710123 China

**Keywords:** Neuropathic pain, Larixyl acetate, Microglia, Neuroinflammation, TRPC6, p38

## Abstract

**Background:**

Neuropathic pain is a debilitating status that is insusceptible to the existing analgesics. It is important to explore the underlying pathophysiological changes and search for new pharmacological approaches. Transient receptor potential canonical 6 (TRPC6) is a mechanosensitive channel that is expressed by dorsal root ganglia and glial cells. It has been demonstrated that this channel in dorsal root ganglia plays essential roles in the formation of mechanical hyperalgesia in neuropathic pain. Recent pharmacological screening suggests that larixyl acetate (LA), a main constituent of larch resin, is able to selectively inhibit TRPC6 function. But whether LA is effective in treating neuropathic pain remains unknown. We investigated the efficacy of LA in rat neuropathic pain model, examined its effects on central neuroinflammation, and explored the possible molecular mechanisms by targeting the spinal dorsal horn.

**Methods:**

Spared nerve injury (SNI) was conducted in Sprague-Dawley rats. Mechanical hypersensitivity and cold allodynia before and after single and multiple *i.t.* applications of LA at the dose of 3, 10, and 30 μM were evaluated by von Frey filament and acetone tests, respectively. Western blot, immunohistochemical, and immunocytochemical stainings were employed to examine the level and expression feature of ionized calcium-binding adaptor molecule 1 (Iba-1), glial fibrillary acidic protein (GFAP), TRPC6, and phosphorylated p38 kinase. The changes of cytokine concentrations, including that of TNF-α, IL-1β, IL-6, and IL-10, were also assessed by multiplex analysis. TRPC6 antisense strategy was finally adopted to investigate the action mechanisms of LA.

**Results:**

Single application of LA on day 5 post injury caused dose-dependent inhibition of mechanical allodynia with the ED_50_ value of 13.43 μM. Multiple applications of LA at 30 μM not only enhanced the analgesic efficacy but also elongated the effective duration without obvious influences on animal locomotor activities. Single and multiple administrations of LA at 30 μM played similar but weaker inhibitory effects on cold allodynia. In addition to behavioral improvements, multiple applications of LA for 6 days dose-dependently inhibited the upregulation of Iba-1, TNF-α, IL-1β, and IL-6, whereas had no obvious effects on the levels of GFAP and IL-10. Combined Western blot and immunostaining assays revealed that the expression of TRPC6 was significantly increased in both spinal dorsal horn after nerve injury and the cultured microglia challenged by LPS, which was however suppressed by the addition of LA at 30 μM or 10 μM, respectively. Further knockdown of TRPC6 with antisense oligodeoxynucleotide produced prominent analgesic effects in rats with SNI, accompanied by the reduced phosphorylation level of p38 in the microglia.

**Conclusions:**

These data demonstrate that *i.t*. applied LA exhibits analgesic and anti-inflammatory action in neuropathic pain. The action of LA involves the suppression of TRPC6 and p38 signaling in the microglia. LA may be thus a promising pharmacological candidate for the treatment of intractable chronic pain.

## Highlights

Intrathecal application of larixyl acetate exerts the analgesic effect on neuropathic pain.

This analgesic effect is associated with the anti-inflammatory action in the spinal cord.

Suppression of the upregulation of transient receptor potential canonical 6 (TRPC6) and pp38 in microglia contributes to the analgesic function of larixyl acetate (LA).

## Background

Neuropathic pain is defined as the pain caused by a lesion or disease of the somatosensory system [1]. The prevalence of neuropathic pain is estimated to be between 1.5 and 8%, equivalent to 100 million and 560 million people worldwide [2]. One notorious clinical manifestation of neuropathic pain is the resistance to treatments with nonsteroidal anti-inflammatory drugs and even opioids [3], implying that the underlying mechanisms are differential from that of the inflammatory pain. Thus, to explore the pathophysiological changes and seek new therapeutic approaches remain core tasks for the preclinical and clinical research.

Neuropathic pain involves the imbalanced expression of a big reservoir of ion channels in primary afferent neurons, such as voltage-gated Na^+^ and Ca^2+^ channels, K^+^ channels, and hyperpolarization-activated cyclic nucleotide-gated channels [3]. These channels either instigate ectopic activity that directly contributes to spontaneous, stimulus-independent pain, or facilitate the release of neurotransmitter and affect the excitability of the central neurons in the spinal cord [2]. Thus, in addition to spontaneous pain, neuropathic pain is also characterized by thermal and mechanical allodynia/hyperalgesia according to the responsive modality of stimuli.

Transient receptor potential (TRP) family encodes nonselective cation channels that sense broad physicochemical stimuli in varied tissues and cell types [4]. TRP family consists of seven subfamilies, canonical (TRPC), vanilloid (TRPV), melastatin (TRPM), ankyrin (TRPA), polycystin (TRPP), mucolipin (TRPML), and TRPN (no mechanoreceptor potential C) [5]. Several members in this family have been demonstrated to be expressed in dorsal root ganglia (DRG) and constitute the possible channel spectrum responsible for thermal transduction in the primary afferent neurons, e.g., TRPV1 (> 42 °C), TRPV2 (> 52 °C ), TPRV3 (~ 39 °C), TRPV4 (> 27 °C), TRPM8 (10~23 °C), TRPA1 (17 °C) [6]. The in vivo functions of these TRP channels, especially that of TRPV1, have been disclosed in varied inflammatory and neuropathic pain models, probably benefited from its much-advanced pharmacological research. It has been shown that TRPV1 is upregulated in DRG following partial nerve injury and spinal nerve ligation [7, 8], and TRPV1 antagonists are effective to inhibit injury-induced hyperalgesia [9]. Our previous research also confirmed the expression feature and in vivo role of TRPV1 in diabetic painful neuropathy [10]. The agonist (capsaicin) and antagonist of TRPV1 have been adopted in clinic or clinical trials for years and remain the promising candidates for the treatment of neuropathic pain [2, 3].

Mechanical allodynia is another feature of neuropathic pain. But so far, the molecules sensing this stimulus and their in vivo functions in neuropathic pain remain elusive. TRPC6 is a Ca^2+^ permeable channel and can be activated by diacylglycerol in a protein kinase C independent manner [11]. TRPC6 is also sensitive to mechanical stimuli such as stretch and hypotonicity [4, 12]. It has been demonstrated that the synergistic activation by receptor and mechanical stimulation exists in TRPC6 via phospholipase C/diacylglycerol and phospholipase A2/ omega-hydroxylase/20-HETE pathway [13]. An essential role of TRPC6 in the mechanical hypersensitivity was investigated by Alessandri-Haber et al. [12]. The author demonstrated that TRPC6 is expressed in DRG neurons and frequently coexisted with TRPC1 and TRPV4, the other two stretch activated ion channels (SAC). Mechanical hyperalgesia can be reversed by intradermal injection of SAC inhibitor, GsMTx-4, or by intrathecal application of oligodeoxynucleotides antisense to TRPC6 [12]. In addition to the role of TRPC6 in DRG, a few recent studies suggest that this channel is also expressed in microglia and astrocytes and is involved in the Ca^2+^ influx that is necessary for their activation following Aβ or lipid challenge [14, 15]. Neuropathic pain also involves overactivity of microglia and, to a less extent, astrocytes in the spinal cord [16], which intensively contributes to the onset and maintenance of central sensitization [17]. It is not yet clear whether TRPC6 contributes to the activation of neuroglia in spinal cord of neuropathic pain.

The lack of specific antagonist and agonist limits the large-scale in vivo study of TRPC6 and hinders thereby the translational research of this channel. A recent study by Urban et al. [18] pointed out that a main constituent of larch resin, larixyl acetate (LA), is able to inhibit the recombinant TRPC6 function and exhibits 12- and 5-fold selectivity compared with its closest relatives TRPC3 and TRPC7. But whether LA is effective in suppression of varied modalities of neuropathic pain is unknown. Based on the previous in vitro study of TRPC6 [19–21], we tested the efficacy of LA on mechanical hypersensitivity and cold allodynia, another modality of neuropathic pain, in a well-established neuropathic pain model, spared nerve injury (SNI). The influences of LA on the activity of neuroglia, the level of cytokines, as well as TRPC6 channel per se and its downstream signaling were also investigated in the spinal cord.

## Methods

### Animals

Adult male Sprague-Dawley rats (220–250 g) were used in the study. The animals were kept at room temperature (RT) with a standard laboratory diet and tap water supplied ad libitum. A 12-h light/dark cycle was maintained during the whole process with light on at 8 AM. All the experiments were conducted in accordance with the Guide from the Animal Use and Care Committee for Research and Education of the Fourth Military Medical University (Xi’an, China, Animal Ethical Committee approval number: FMMU-AEEA-20170404) and the IASP’s guidelines for pain research [22].

### Intrathecal implantation

Rats were deeply anesthetized with 7% chloral hydrate (4 mL/kg, *i.p.*). The 5th and 6th lumbar spinous processes were exposed and the 6th was removed. A PE-10 tubing filled with saline was inserted from interspinal interstice into the subarachnoid space. One tip of the catheter was passed to reach the lumbar enlargement of the spinal cord, and another tip was tightly tied around the lumbar tissue and implanted subcutaneously at the back of the neck. Ten microliter of 2% lidocaine followed by the same volume of saline was injected into the catheter on the following day, to test if the operation was successful. Only rats showing complete paralysis of both hind limbs were used for the further study. The position of the tubing was verified after each sacrifice. Data from those with incorrect tubing were discarded.

### Neuropathic pain model induced by SNI

**Fig. 1:**
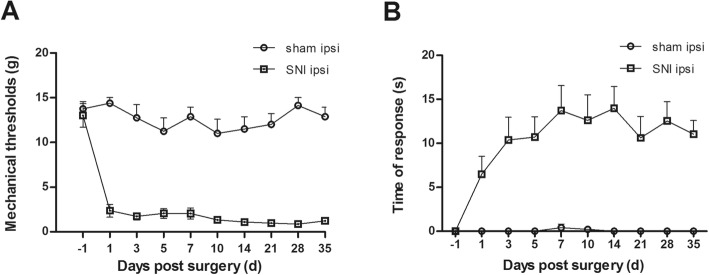
The establishment of neuropathic pain model induced by SNI. **a** The manifestation of the mechanical sensitivity before and after surgery in nerve injured and sham groups. Mechanical thresholds were acquired by von Frey filament test. SNI caused dramatic increase of mechanical sensitivity in the ipsilateral hindpaw from postoperative day (POD) 1 and lasted throughout the follow-up. *n* = 8. **b** Illustration of cold sensitivity in SNI and sham groups. SNI rats developed remarkable response to acetone application in the ipsilateral side since POD 1, while this response was actually missing in the sham group. *n* = 8

After recovery for 3 days, the rats were anesthetized again (7% chloral hydrate, 4 mL/kg,* i.p.*) and SNI was produced according to the method described by Decosterd and Woolf [23]. Briefly, after incision of skin at the middle of the left thigh, the sciatic nerve and its three terminal branches were exposed by blunt dissection through the biceps femoris. The tibial and common peroneal nerves were tightly ligated with 5.0 silk and sectioned distally. A nerve segment of ~ 3 mm distal to the section was additionally removed. During the procedure, great care was taken to maintain the sural nerve intact. The sham animals underwent the exposure of the sciatic nerve without further operations. For the long-term observation of pain behaviors as illustrated in Fig. 1, normal rats instead of catheterized rats were used for SNI or sham operations.

### Drug administration

**Fig. 2:**
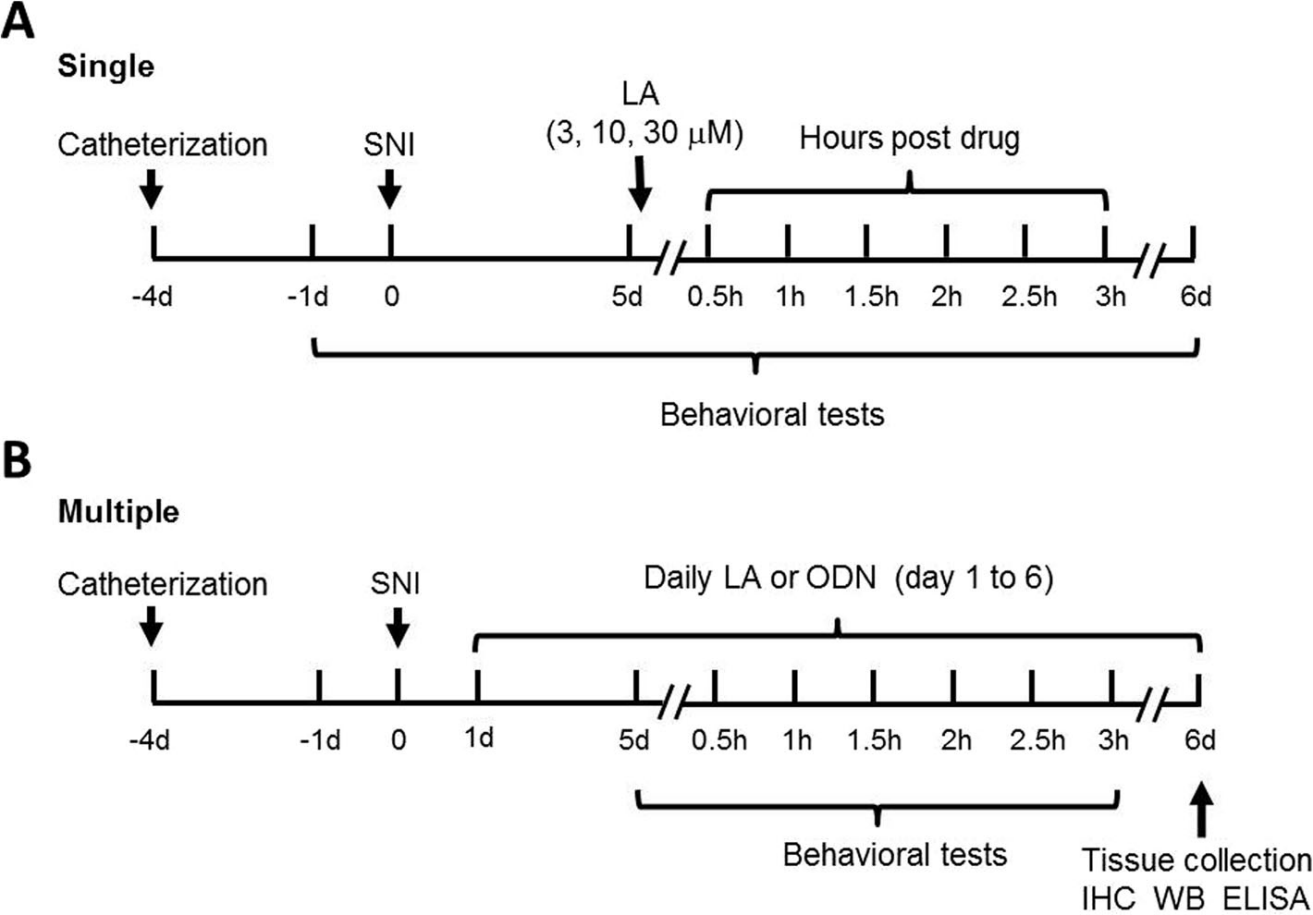
Flow chart illustrating two experimental paradigms. **a** Single application. The catheterization was performed 4 days prior to SNI operation. LA at 3, 10, 30 μM was *i.t.* applied on day 5 post nerve injury. Mechanical and thermal tests were conducted 1 day prior to nerve injury and on POD 5 and 6. After drug application, mechanical tests were elaborately performed six times within 3 h, i.e., every 30 min a time. The cold allodynia was observed every 1 h, totally three times. **b** Multiple applications. The operation schedule for catheterization and SNI was the same as the single paradigm. LA at 30 μM, TRPC6 antisense or mismatch ODNs with or without supplementation of LA at 30 μM was *i.t.* applied daily from day 1 to 6 after SNI. Behavioral tests were performed pre- and post-drug application on day 5. On day 6, the drug was applied again and rats were sacrificed around 2.5 h post application after the final behavioral observation. Tissue collections for immunohistochemical (IHC) staining, Western blot (WB) and multiplex measurement (ELISA) were performed thereafter

LA, purchased from Toronto Research Chemicals Inc. (L175955, Toronto, Canada), was dissolved in chloroform at 10 mM and diluted in saline to concentrations of 3, 10, 30 μM. For the single application (Fig. 2a), the sham or SNI rats on postoperative day (POD, nominated after nerve injury) 5 were gently handled and 10 μL of LA at varied doses followed by 10 μL of saline was injected into the subarachnoid space of lumbar enlargement. The rats were then subjected to behavioral tests at 0.5, 1, 1.5, 2, 2.5, 3 h and on post day 1 (POD 6) to estimate the drug effects. Then, 0.3% chloroform (the highest concentration of chloroform in solution) in saline was used as vehicle. In addition to this acute paradigm, LA at 30 μM was also repetitively applied (Fig. 2b) for consecutive 5 days from POD 1 to 5 to observe the long-term behavioral effects and applied for another 1 day on POD 6 before sacrificing and harvesting the tissue.

### Preparation and administration of oligodeoxynucleotide for TRPC6

The sequences for antisense and mismatch oligodeoxynucleotide (ODN) were adopted from the literature [12] and were synthesized from Sangon Biotech (Shanghai, China). Briefly, the TRPC6 antisense (5′-ATAGTCCTGGCTCTCGTTGC-3′) was directed against a unique region of the rat TRPC6 protein (GenBank accession number NM-053559). The mismatch sequence represents the mismatching of eight bases of TRPC6 antisense (5′-**TATC**TCCT**C**GCTCTC**CAA**GC-3′, denoted in bold). For the sole application, ODN was reconstituted in nuclease-free 0.9% NaCl and was *i.t.* applied at the dose of 40 μg/10 μL followed by 10 μL of saline, once per day for 5 days. For the co-administration, 10 μL of LA at 30 μM was subsequently applied after each ODN delivery. Behavioral tests were performed at approximately 2.5 hours post application to comply with the pharmacokinetics of LA. Likewise, after final delivery on POD 6, the tissue of the spinal cord was harvested for Western blot and immunohistochemical staining.

### Behavioral testing

#### Mechanical sensitivity

Mechanical allodynia was detected using von Frey filament tests as described previously [24]. The rats were placed in the transparent box with an elevated metal mesh floor and were allowed to acclimate for 30 min before testing. The mechanical withdrawal threshold was measured at the left hind paw with von Frey hairs stimulation by the up-down method. The lateral plantar surface of the hind paws (the territory of the sural nerve) was perpendicularly stimulated with a series of von Frey hairs with logarithmically increasing stiffness (0.04–10 g). Positive responses were defined as a sharp withdrawal or flinch of the hind paw following filament application. The weakest force (g) to induce positive response at least three times in five trials was referred to as the paw withdrawal threshold. To avoid unnecessary skin damage, the value was recorded as 15 g if the response at 10 g was negative. For long-term observations (Fig. 1a), the mechanical allodynia was measured at 1 day prior to and 1, 3, 5, 7, 10, 14, 21, 28, 35 days post neuropathy. All behavioral tests were performed in a blinded manner.

#### Cold allodynia

**Fig. 3:**
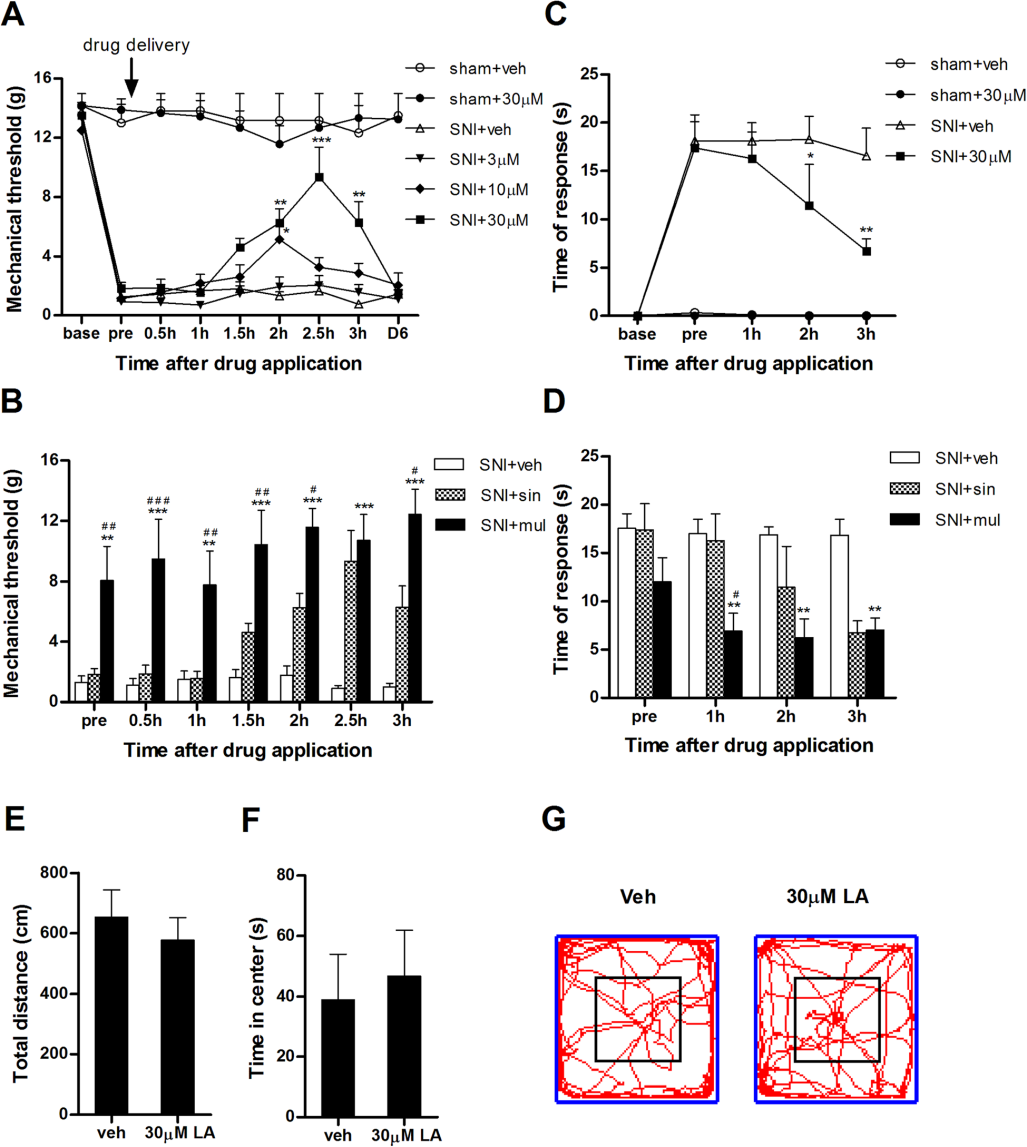
The analgesic action of LA after single or multiple applications. **a** Single application of LA at 3, 10, and 30 μM on POD 5 dose-dependently alleviated mechanical allodynia. The effect of LA at 10 μM in rats with SNI was transiently observed at 2 h after *i.t.* application. The action of LA at 30 μM lasted longer and was maintained to 3 h. Vehicle (0.3% chloroform) or 30 μM of LA had no marked influences on mechanical response of sham-operated rats. **b** Mechanical test on POD 5 before (“pre”) and after the fifth application of LA at 30 μM. Multiple applications not only enhanced the analgesic efficacy but also elongated action duration. The data for SNI + single (sin) group were adopted from (**a**). **c** Cold sensitivity on POD 5 before and after application of acetone. Acute *i.t.* administration of LA at 30 μM gradually relieved cold allodynia induced by SNI but did not cause visible effects on sham animals. **d** Multiple applications of LA at 30 μM advanced the onset time to 1 h post drug but did not significantly elongated the action duration to the second day. The data for SNI + single group were adopted from (**c**). **e**–**g** Open field test showing that multiple applications of LA in catheterized normal rats did not cause marked influences on animal locomotive activities. **e** Comparison of the total travel distance in 5 min between vehicle and LA (30 μM) treated groups. **f** Time spent in central arena (25% area) of the open field. **g** Typical locomotive traces in two groups. *p < 0.05, **p < 0.01, ***p < 0.001 vs. SNI + veh group. ^#^*p* < 0.05, ^##^*p* < 0.01, ^###^*p* < 0.001 vs. SNI + sin group. *n* = 6–8

To measure cold sensitivity, ~ 100 μL of acetone was applied to the lateral plantar surface of the rat hind paw with the help of a 1 mL syringe, making sure that only the acetone touched the paw and not the tip of the syringe. In the single paradigm, the time rat spent in lifting, shaking, or licking the treated paw during 30 s was recorded with a stop watch before nerve injury (“base” in Fig. 3c), before drug administration (“pre” in Fig. 3c) and at 1, 2, and 3 h post application on POD 5. In the multiple paradigm, the cold test was performed on POD 5 before (“pre” in Fig. 3d) and at 1, 2, and 3 h post interday application. The trial at each time point was performed three times with an intertrial interval of 5 min. Values of most approximate two were averaged and recorded as “time of response”.

#### Open field test

The open field test (OFT) is usually used to evaluate the locomotor, exploration, and anxiety in animals. At present, it was performed in a lusterless perspex box (100 cm × 100 cm × 40 cm). The apparatus was thoroughly cleaned with 75% ethanol and dried before the introduction of each animal. After the final administration of LA at 30 μM, rats were placed in the center of the open field and allowed to explore the apparatus for 5 min. The behaviors of the rats were recorded by a video camera suspended approximately 250 cm above the open-field arena. Two parameters were summarized to assess the locomotive performance: total travel distance and time spent in the central zone (corresponding to 25% area of the total field).

### Western blot analysis

Rats subjected to behavioral tests on POD 6 were anesthetized with chloral hydrate and rapidly sacrificed. The lumbosacral spinal cord was puffed out with a pipette filled with saline. The left spinal dorsal horn (SDH) was dissected on ice and kept in − 80 °C freezer until use. After finishing collection, all tissues were homogenized in SDS sample buffer (10 μL/mg tissue) supplemented with inhibitors of proteinase and phosphatase (Sigma, MO, USA). The homogenate was centrifuged at 4 °C for 10 min at 10000×*g* and the protein concentration was detected by the bicinchoninic acid method. The supernatant was kept in two vials, one for Western blot assay and the other for multiplex measurement. Those for Western blot were heated in boiling water for 5 min and loaded onto SDS-polyacrylamide gels for electrophoresis. The proteins were then transferred onto the polyvinylidene difluoride membrane (Millipore, Billerica, MA, USA). After being blocked in solution containing 5% nonfat milk and Tris-buffered saline with 0.02% Tween-20 (TBST) for 1 h at RT, the membranes were incubated overnight at 4 °C with following primary antibodies: goat anti-Iba-1 (1:200; Abcam, Cambridge, England), rabbit anti-GFAP (1:400; Abcam), rabbit anti-TRPC6 (1:200; Alomone, Jerusalem, Israel), rabbit anti-p38 (1:200; Cell Signaling, Danvers, MA, USA), rabbit anti-phosphorylated p38 (pp38, 1:200; Cell Signaling), and mouse anti-β-actin (1:5000; Sigma). Following complete rinse with TBST, the membranes were next incubated with horseradish peroxidase-conjugated secondary antibodies for 1 h at RT (anti-goat, 1:5000; anti-rabbit, 1:5000; anti-mouse, 1:5000; ZSGB-Bio, Beijing, China). The reactions were detected with enhanced chemiluminescence (ECL) method and images were acquired with the Bio-Rad imaging system. For analysis, ImageJ (NIH software) was used and the levels of target proteins were normalized to those of β-actin and expressed as fold changes relative to the sham + vehicle or mismatch group.

### Multiplex measurement of cytokines levels

Concentrations of interleukin (IL)-1, IL-6, IL-10, and tumor necrosis factor-α (TNF-α) were simultaneously quantified in each spinal cord sample using Milliplex Map rat cytokine kit (Recytmag-65 K-04, Merck, St. Louis, USA). The measurements involved a cocktail incubation of the tissue supernatant with the antibodies for the four analytes, which are immobilized to a set of magnetic beads developed according to Luminex xMAP technology. The detection of the analytes was achieved by sequential incubation with biotinylated antibodies and streptavidin-phycoerythrin. The reporter fluorescent signal was measured with a Bio-Plex 200 reader (Bio-Rad, Hercules, CA, USA). Data were calculated with a calibration curve obtained in each experiment using recombinant standard cytokines diluted in kit assay buffer and lysis buffer. Concentrations of cytokines were finally acquired using Milliplex analyst software (VigeneTech, Carlisle, MA, USA) with a five-parameter logistic curve-fitting method.

### Immunohistochemical staining

Following pain tests on POD 6, rats were deeply anesthetized and perfused transcardially with 100 mL of 0.9% saline, followed by 500 mL of 0.1 M phosphate buffer (PB, pH 7.4) containing 4% (w/v) paraformaldehyde. The spinal cords were removed, postfixed in the same fixative for 2 h, and cryoprotected overnight at 4 °C in 0.1 M PB with 30% (w/v) sucrose. The lumbosacral segments of the spinal cords were cut at 30 μm in thickness in a cryostat (CM1950; Leica, Heidelberg, Germany). The sections were rinsed in 0.01 M phosphate-buffered saline (PBS, pH 7.2) and blocked with 10% fetal bovine serum in 0.01 M PBS for 1 h at RT.

For pictures illustrated in Figs. 5 and 6, the sections were then sequentially incubated with (1) rabbit anti-Iba-1 antibody (1:1000; Wako, Osaka, Japan) or mouse anti-GFAP antibody (1:4000; Millipore) at 4 °C for 48 h; (2) biotinylated donkey anti-rabbit or anti-mouse antibody (1:500; Millipore) for 6–8 h at RT; and (3) avidin-biotin complex in ABC elite kit (1:200; Vector, CA, USA) for 2 h at RT. Between the steps, 0.01 M PBS was used to thoroughly rinse the sections. The incubation medium used for the primary and secondary antibodies was 0.01 M PBS (pH 7.4) containing 2% normal donkey serum, 0.3% Triton X-100, 0.25% λ-carrageenan, and 0.05% sodium azide. Finally, the sections were incubated in buffer containing 3,3′-diaminobenzidine tetrahydrochloride and H_2_O_2_ to visualize the Iba-1 or GFAP-positive staining. The sections were then mounted onto gelatin-coated glass slides, dehydrated in ethanol, vitrified in dimethylbenzene, and coverslipped with DPX mountant. The staining was observed under Olympus BX60 microscope (bright field mode) and digital images were captured with Olympus DP70 camera. The gray value of the dorsal horn in different groups was analyzed with a NIH free software ImageJ. Every group comprised three rats and three to five sections (L4-L5 level) from each rat were included in the analysis. The relative averaged gray values in the ipsilateral side against that of contralateral side were finally adopted to eliminate individual differences (Figs. 5h and 6h). The specificity of immunostaining was tested by omitting the primary antibodies and the results were negative and not shown.

For immunofluorescent staining in Figs. 8b and 9d, sections were sequentially incubated with rabbit anti-TRPC6 antibody (1:200, Alomone), biotinylated donkey anti-rabbit antibody (1:500; Millipore), and Alexa Fluor 488 conjugated avidin (1:1000; Invitrogen, Carlsbad, CA, USA). To test the specificity of TRPC6 antibody, equal amount of antigen provided by the manufacturer was used to pre-absorb the antibody before the incubation with sections. For double-labeled immunofluorescent staining as illustrated in Fig. 10, sections were sequentially incubated with rabbit anti-pp38 (1:200; Cell Signaling) and goat anti-Iba-1 (1:200; Abcam), biotinylated donkey anti-rabbit antibody (1:500; Millipore), and Alexa Fluor 594 conjugated donkey anti-goat antibody (1:500; Invitrogen), Alexa Fluor 488 conjugated avidin (1:1000; Invitrogen). The incubation medium, duration, and interstep procedures were exactly the same as the above-mentioned immunohistochemical staining. After being mounted and coverslipped, the sections were observed under a confocal laser scanning microscope (FV-1000, Olympus, Tokyo, Japan) with the appropriate laser beams and filter settings for Alexa Fluor 488 (excitation: 488 nm; emission: 510–530 nm) and Alexa Fluor 594 (excitation: 543 nm; emission, 590–615 nm). Digital images were captured and analyzed using a Fluoview 1000 (Olympus). For intergroup comparisons in Figs. 8b and 10, the capture settings and subsequent image analysis were kept exactly the same.

### Cell culture and immunocytochemical staining

Microglial cells were isolated from the cerebral hemispheres of neonatal mouse brains (P1–2). Briefly, after being dissected and removed of the skull and meninges under a microscope, the cortical tissue was minced and digested with 0.125% trypsin. Cells were plated onto poly-l-lysine (PLL)-coated tissue culture T-75 flasks and allowed to grow for 10–14 days in Dulbecco’s modified Eagle medium (DMEM) (Gibco, Life technologies, Carlsbad, CA, USA) containing 10% FBS and 2 mM l-glutamine. Microglial suspensions were then collected by shaking the flasks at 220 rpm for 3–6 h. After centrifugation at 800 × *g* for 5 min, cells were resuspended in DMEM with 10% FBS and plated onto PLL-coated 6-well plates at a density of 2 × 10^5^ cells/cm^2^. In this way, the purity of microglia can reach more than 95% based on staining with the Iba-1 or F4/80 antibody before use.

When the purified microglial cells reached 80% confluency, four differential treatments were applied as follows: normal, incubation with lipopolysaccharide (LPS) at 500 ng/mL for 24 h, incubation with LPS plus 10 μM or 30 μM of LA for 24 h. After being rinsed with PBS, the cells were fixed with 4% paraformaldehyde for 20 min and blocked with 5% bovine serum albumin and 0.3% Triton-100 in 0.01 M PBS for 1 h at RT. The cells were then subjected to the following incubation successively: rabbit anti-TRPC6 antibody (1:200, Alomone) and goat anti-Iba-1 (1:200; Abcam) overnight at 4 °C; Alexa Fluor 488 conjugated donkey anti-rabbit antibody (1:500; Invitrogen) and Alexa Fluor 594 conjugated donkey anti-goat antibody (1:500; Invitrogen) for 2 h at RT; 4′,6-diamidino-2-phenylindole (DAPI, 1:1000; Sigma) for 10 min at RT. Thorough PBS rinsing was performed between each two steps. The images were captured under the confocal laser scanning microscope (FV-1000, Olympus). Again, pre-absorption of TRPC6 antibody was performed to assess the specificity of the staining.

### Statistical analysis

The data are expressed as means ± standard error of the means (SEM). The normality of data was tested using the D’Agostino and Pearson omnibus normality test. The data from Western blot assays and multiplex measurements were analyzed by one-way analysis of variance (ANOVA) followed by Dunnett’s multiple comparison test. Those from behavioral observations at different time points were analyzed by two-way ANOVA followed by Bonferroni’s multiple comparison test. All these statistical analyses were performed with GraphPad Prism version 5.01 for Windows (Graph Pad Software, San Diego California, USA). A *p* value < 0.05 was considered significant.

## Results

### Establishment of the neuropathic pain model induced by SNI

After operation, animals in the sham group showed fluctuated but overall stable mechanical thresholds around 12 g in the ipsilateral hind paw from POD 1 to POD 35 (Fig. 1a; *n* = 8). In contrast, rats in the SNI group developed a marked hypersensitivity to the stimulation applied with von Frey filament since POD 1 (Fig. 1a; *n* = 8). The ipsilateral mechanical threshold was maintained at 1–2 g throughout the observation period. Meanwhile, rats with SNI also exhibited intense responses such as lifting, shaking, and/or licking the ipsilateral paw to the acetone application since POD 1. By contrast, sham rats showed almost null response to the acetone application during the whole observation period (Fig. 1b; *n* = 8). Notably, neither mechanical nor cold sensitivity was strikingly changed in the contralateral side of SNI rats before and after nerve injury (data not shown). These behavioral results demonstrated the successful establishment of the rat neuropathic pain model induced by SNI.

### The analgesic action of LA after single and multiple applications

#### On mechanical hypersensitivity

**Fig. 4:**
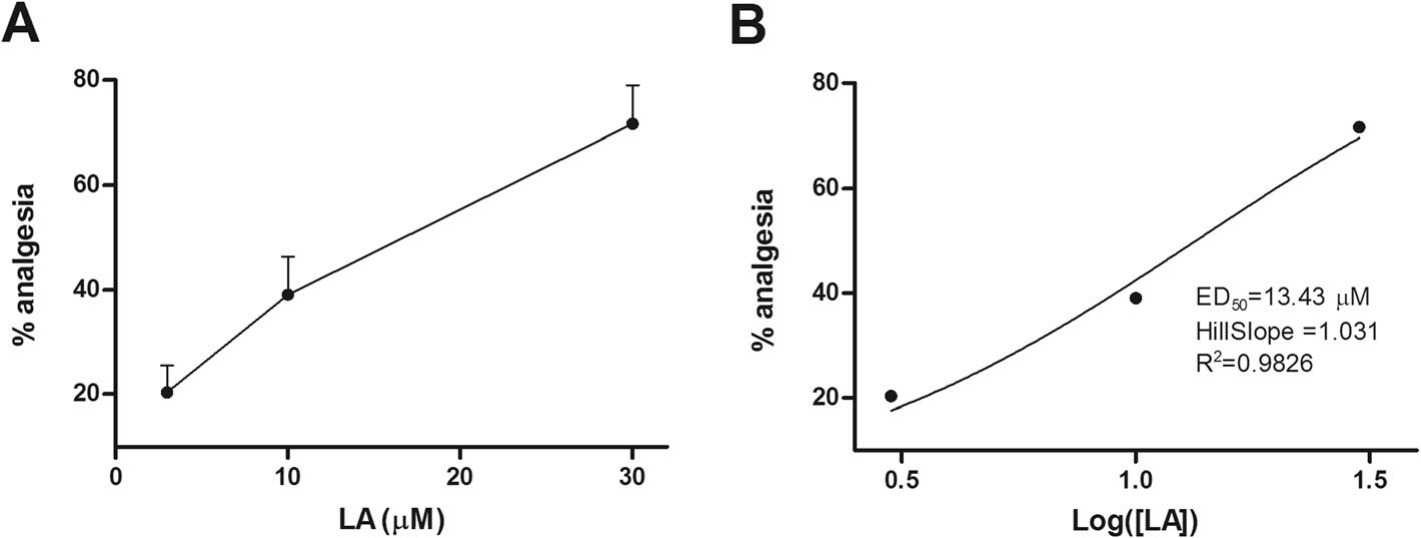
Dose-response relationship for the analgesic effects of LA. **a** Linear plot of the anti-nociceptive effects of LA at 3, 10, and 30 μM. The analgesia denotes the percentage of the highest mechanical thresholds after drug application to the thresholds prior to nerve injury in SNI group. *n* = 6–8. **b** Nonlinear regression of the data in A. The ED_50_, HillSlope and *R*^2^ were automatically calculated using GraphPad Prism 5 software

In the single paradigm (Fig. 2a), *i.t.* administration of vehicle or LA at 30 μM had no detectable influences to the mechanical thresholds of the sham rats on POD 5 (Fig. 3a). However, delivery of LA at 3, 10, or 30 μM dose-dependently inhibited pain responses of SNI rats within 3 h of observation. As shown in Fig. 3a, application of 3 μM of LA caused no obvious changes to the mechanical threshold of SNI rats compared with vehicle treated group. In contrast, 10 μM of LA significantly lifted the mechanical threshold to 5.13 ± 0.96 g (*p* < 0.05 vs. SNI + veh, *n*=7) at 2 h after drug, although the effect quickly disappeared. Single application of LA at 30 μM advanced the onset of the drug to 1.5 h but with no statistical significance. The efficacy however turned stronger at 2 h, reached the apex at 2.5 h (9.33 ± 2.03 g, *n*=6), and slightly declined at 3 h. Nevertheless, the mechanical thresholds at the three time points were all significantly higher than that of the vehicle group (*p* < 0.01, *p* < 0.001, *p* < 0.01 vs. SNI + veh, respectively). The analgesic effect of LA at 30 μM disappeared on the second day, i.e. POD 6 before the final application. These results demonstrated that single application of LA at 10 and 30 μM was effective to relieve mechanical allodynia in rats with neuropathic pain. For more accurate appraisal, we built the dose-response curve of LA and simulated it with nonlinear regression (Fig. 4). It revealed that the analgesic effect of LA was perfectly fitted and the calculated ED_50_ value was 13.43 μM.

We next employed multiple paradigm (Fig. 2b) to explore the long-term effects of the drug. As summarized in Fig. 3b, consecutive application of LA at 30 μM for 5 days significantly alleviated the mechanical allodynia of SNI group at all observed time points compared with multiple treated vehicle group (*p* < 0.01 or 0.001 vs. SNI + veh, *n*=7-8). Notably, the pain threshold on POD 5 prior to the intraday application was also significantly higher than that of the vehicle group ("pre" columns in Fig. 3b; *p* < 0.01 vs. SNI + veh group), suggesting that the effect of LA could be maintained to the second day after applications for four times. In addition, the analgesic effects of LA produced by long-term application were also significantly stronger than that by single application of LA at all time points except for 2.5 h, suggesting that this time point may be suitable for the stable observation of the analgesic effects irrespective of the application paradigm. We therefore chose to sacrifice animals for tissue collection at this time point after the final drug application and behavioral observation on POD 6.

#### On cold hypersensitivity

Neuropathic pain is also characterized by the increased sensitivity to thermal stimulus. We observed the effects of LA at 30 μM, the most effective dose in mechanical test, on cold allodynia. As shown in Fig. 3c, single *i.t*. application of LA at this dose had no obvious effects on cold response in sham animals compared to vehicle-treated group throughout the observation. However, it caused fluctuated but significantly reduced response at 2 h and further alleviating and much uniform effects at 3 h in SNI rats compared with the vehicle treated SNI group (Fig. 3c; *p* < 0.05, *p* < 0.01 vs. SNI + veh respectively, *n* = 6). Multiple applications of LA advanced the onset of the drug to 1 h and maintained the effects during the following 2 h. It is noteworthy that the cold response on POD 5 prior to the intraday application was visibly but insignificantly lowered than that of the vehicle group (“pre” columns in Fig. 3d), suggesting that the effect of LA on cold allodynia could not be well maintained to the second day after applications for four times.

#### On locomotor activities

To examine whether LA affects the locomotive activities after multiple applications, normal rats with preceding intrathecal catheterization were assessed in open field test. As shown in Figs. 3e–g, chronic application of LA at 30 μM neither affected the total travel distance nor influenced the time spent in central arena compared with the vehicle treated group, suggesting that multiple *i.t.* applications of LA do not cause obvious effects on animal locomotive behaviors (*p* > 0.05, *n* = 6–8).

### The influences of LA on glia and neuroinflammation

#### The inhibitory role in Iba-1 expression

**Fig. 5:**
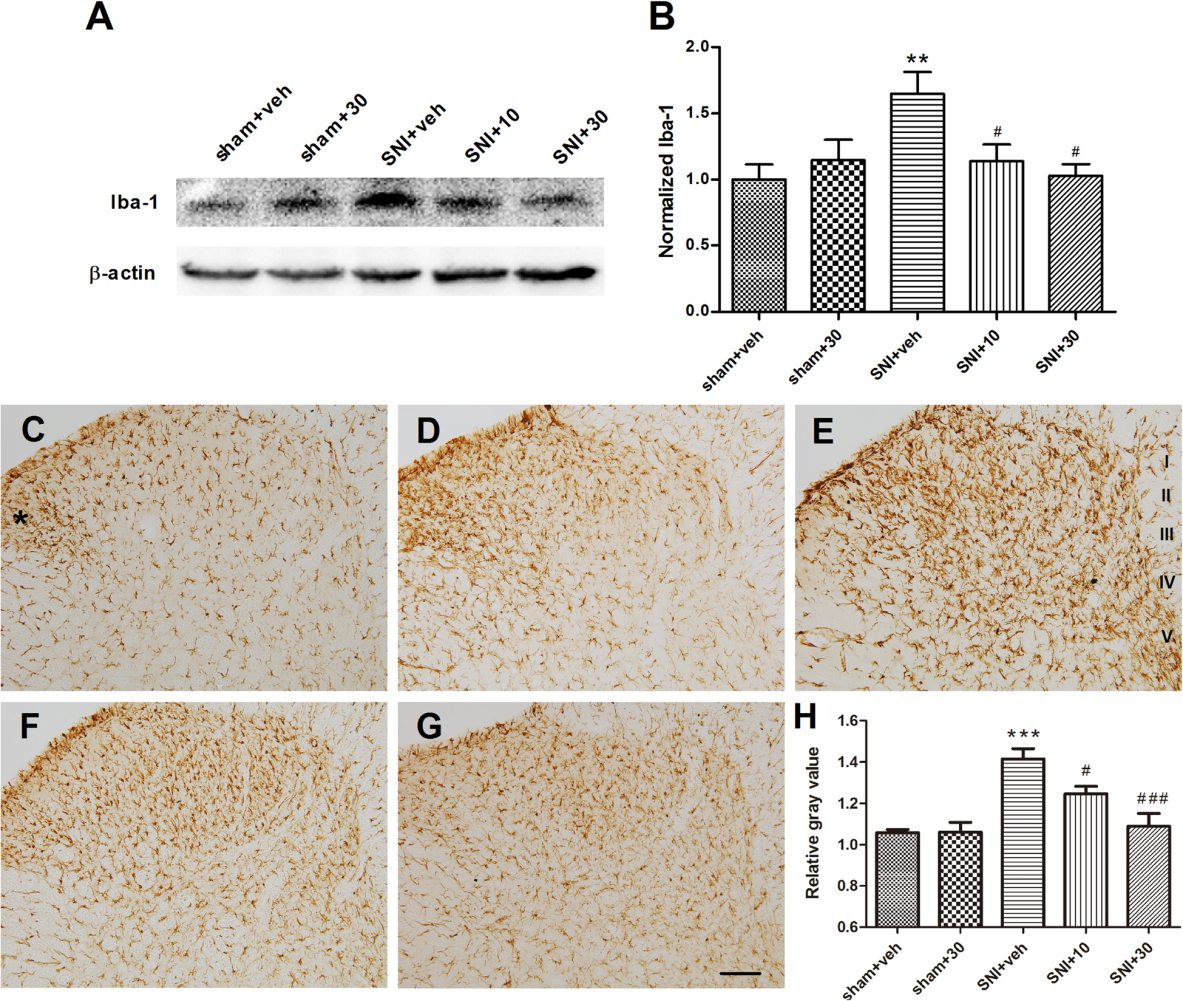
The effects of repeated applications of LA on the expression of Iba-1 in the ipsilateral SDH on POD 6. **a**, **b** Western blot analysis showing that LA dose-dependently suppressed the elevated Iba-1 induced by SNI. **a** Typical blotting trace. **b** Summary of the blotting results. *n* = 5. **c**–**g** Immunohistochemical staining showing the distribution of Iba-1 IR in five groups. **c** Typical picture in a vehicle treated sham rat. Iba-1-positive microglia were evenly distributed in the SDH, except for the lateral border of the superficial dorsal horn (marked by asterisk). **d** Sham group treated with 30 μM of LA. **e** Vehicle-treated SNI group, showing that nerve injury caused strong detection of Iba-1 IR in the medial two thirds of SDH, mainly laminae I-V. **f**, **g** Typical Iba-1 staining in SNI rats treated with 10 μM (**f**) or 30 μM (**g**) of LA, showing that *i.t.* application of LA markedly repressed the intensity of Iba-1 IR. Scale bar, 100 μm. **h** Summary of averaged gray value for Iba-1 staining in the SDH in five groups. Relative gray value was calculated as the ratio of averaged gray value in the ipsilateral side (mainly covering laminae I-V) to that in the contralateral side. *n* = 3. **p < 0.01, ***p < 0.001 vs. sham + veh group; ^#^*p* < 0.05, ^###^*p* < 0.001 vs. SNI + veh group

Microglia undergo intensive activation following neuropathic pain. We investigated whether long-term application of LA influenced the expression of Iba-1, a specific marker for microglia activation. Western blot assay showed that SNI on POD 6 induced significant increase of Iba-1 expression in the ipsilateral dorsal horn compared to the sham group (Fig. 5a, b; *p* < 0.01 vs. sham + veh, *n* = 5). Application of LA at the dose of 10 μM for 6 days significantly inhibited this tendency and that at 30 μM reversed the increase to a level indiscriminate to that of sham group (Fig. 5a, b; *p* < 0.05 vs. SNI + veh, *n*=5). Of note, long-term treatment with LA at 30 μM had no obvious influence on the level of Iba-1 in the sham group compared to the vehicle-treated one.

Further immunohistochemical staining showed that Iba-1 immunoreactivities (IR) in the vehicle and 30 μM of LA-treated sham groups were not evenly distributed in the ipsilateral dorsal horn, with much stronger staining appeared at the lateral border of superficial dorsal horn than that in the remaining dorsal horn (Fig. 5c, d). Nevertheless, such pattern was not different from those in the contralateral side (data not shown), suggesting that they were not resulted from operation. In contrast, nerve injury on POD 6 caused condensed and strong detection of Iba-1 IR in the medial two thirds of the superficial (laminae I and II) and deep dorsal horn (laminae III-V), suggestive of the microgliosis in these extensive areas (Fig. 5e). This change was not observed in the contralateral side (data not shown). Repeated applications of LA at both 10 and 30 μM essentially inhibited the strong Iba-1 IR in the ipsilateral dorsal horn induced by SNI (Fig. 5f, g). As summarized in Fig. 5h, the relative gray value of Iba-1 staining in the ipsilateral dorsal horn was about 50% stronger in the nerve injured group than that in the sham group (*p* < 0.001 vs. sham + veh, *n* = 3), which was however dose-dependently inhibited by the multiple applications of LA at 10 and 30 μM.

#### The non-influence on GFAP expression

**Fig. 6:**
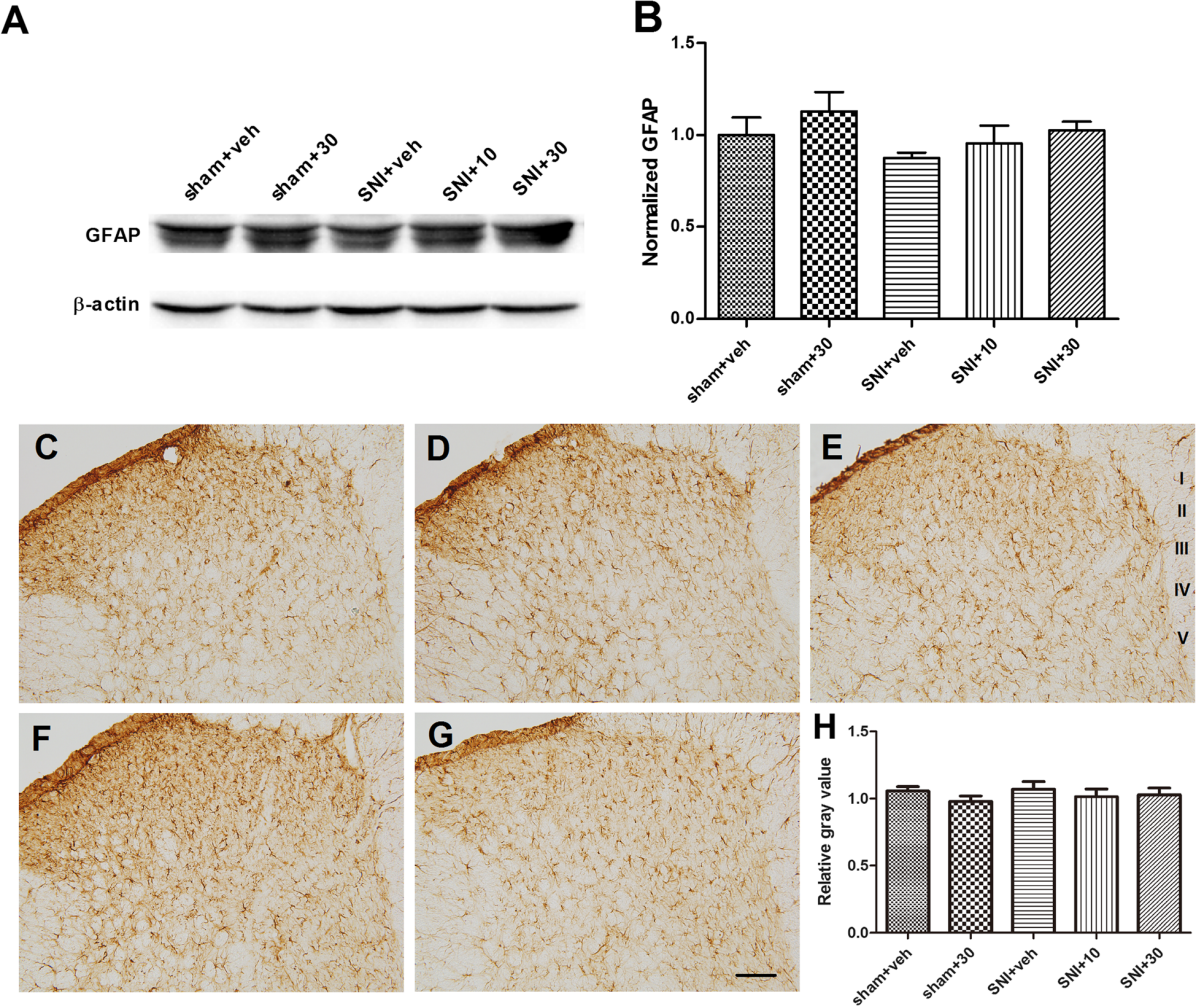
The effects of repeated applications of LA on the expression of GFAP in the ipsilateral SDH on POD 6. **a**, **b** Western blot analysis showing that the level of GFAP was not significantly influenced by either nerve injury or LA treatment. **a** Typical blotting trace. **b** Summary of the blotting results. *n* = 5. **c**–**g** Typical immunohistochemical staining showing the distribution of GFAP IR in the SDH in five groups. **c** Sham + vehicle group; **d** sham + LA (30 μM) group; **e** SNI + vehicle group; **f** SNI + LA (10 μM) group; **g** SNI + LA (30 μM) group. GFAP positive astrocytes were much densely distributed in the superficial dorsal horn in the sham groups (**c**, **d**). Both the density and morphology of the cells were not obviously affected by the nerve injury or LA treatment (**e**–**g**). Scale bar, 100 μm. **h** Summary of gray value for GFAP staining in five groups. Relative gray value was calculated the same as that of Iba-1. *n* = 3

Peripheral inflammation or nerve injury can also cause astrocyte gliosis. We therefore examined the change of GFAP and the influence of LA under the same paradigm. As shown in Fig. 6a, b, Western blot analysis revealed that the expression of GFAP, the marker of astrocyte, was not significantly changed in the ipsilateral dorsal horn among all the five groups (*p* > 0.05, *n* = 5). Further immunohistochemical staining demonstrated that GFAP IR were more densely located in the superficial dorsal horn (laminae I to II) and lamina III in vehicle-treated sham group (Fig. 6c). Within this region, however, the cells were relatively evenly distributed along the mediolateral axis, which was different from those in Iba-1 staining (Fig. 6c). Astrocytes in the deep dorsal horn were relatively sparsely distributed. Long-term application of LA at 10 and 30 μM caused no detectible changes to the aforementioned intensity and distribution feature of astrocytes in sham and SNI groups (Fig. 6d–g). Further gray value analysis confirmed the observed phenomenon and verified the data from Western blot analysis (Fig. 6h; *n*=3).

#### The downregulation of proinflammatory cytokines

**Fig. 7:**
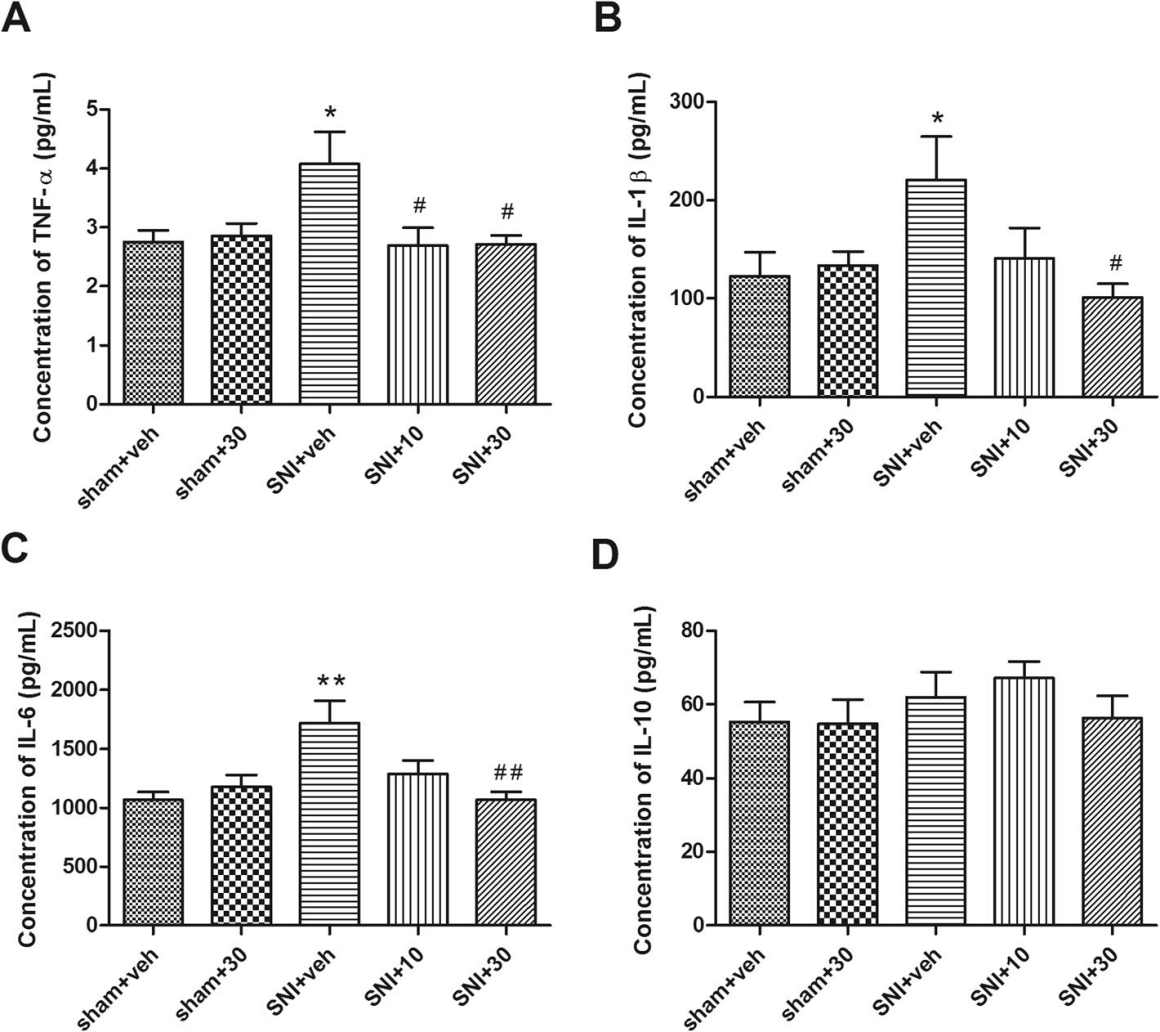
The effects of repeated applications of LA on the levels of cytokines in the ipsilateral SDH. Multiplex assay was employed to simultaneously detect the concentrations of four cytokines in each particular sample. **a** The concentration of TNF-α in the five groups with differential treatments. LA at 10 and 30 μM both significantly suppressed the upregulation of TNF-α induced by nerve injury. **b** The concentration of IL-1β in the five groups. LA at 30 μM other than 10 μM significantly reversed the increased level of IL-1β in the SNI group. **c** The alteration of IL-6 in the five groups was similar to that of IL-1β. **d** The level of IL-10 was not statistically different among the five groups. *p < 0.05, **p < 0.01 vs. sham + veh group; ^#^*p* < 0.05, ^##^*p* < 0.01 vs. SNI + veh group. *n* = 5

Neuroinflammation characterized by the dysregulated cytokine expression is frequently reported in varied neuropathic pain models. We investigated the changes of TNF-α, IL-1β, IL-6, and IL-10 induced by nerve injury and the influence of LA on them. As shown in Fig. 7a, the level of TNF-α reached to 4.07 ± 0.55 pg/mL in the ipsilateral dorsal horn of vehicle-treated SNI animals, which was significantly higher than that in vehicle treated sham group (2.75 ± 0.19 pg/mL) (*p* < 0.05, *n* = 5). Multiple treatments with LA at 10 and 30 μM both strikingly reversed the increased level of TNF-α, suggesting that TNF-α is susceptible to the treatment with LA even at relatively low concentration. Similarly, SNI rats on POD 6 also exhibited significantly enhanced IL-1β and IL-6 in the ipsilateral dorsal horn compared with their respective sham groups (Fig. 7b, c; 122.60 ± 24.48 pg/mL vs. 220.80 ± 44.36 pg/mL for IL-1β, *p* < 0.05; 1068 ± 63.43 pg/mL vs. 1715 ± 189.60 pg/mL for IL-6, *p* < 0.01; *n* = 5). Repeated treatment with LA at 10 μM caused visible but insignificant reduction of these two cytokines, which was markedly magnified by LA at 30 μM. By contrast to the changes of proinflammatory cytokines, the levels of IL-10 among five groups were not statistically different from each other, with the levels maintained at the range of 54.78 ± 6.57 pg/mL to 67.19 ± 4.53 pg/mL (*p* > 0.05, *n* = 5). These results demonstrated that LA dose-dependently improved the spinal neuroinflammation induced by nerve injury.

### The expression of TRPC6 and influence of LA

**Fig. 8:**
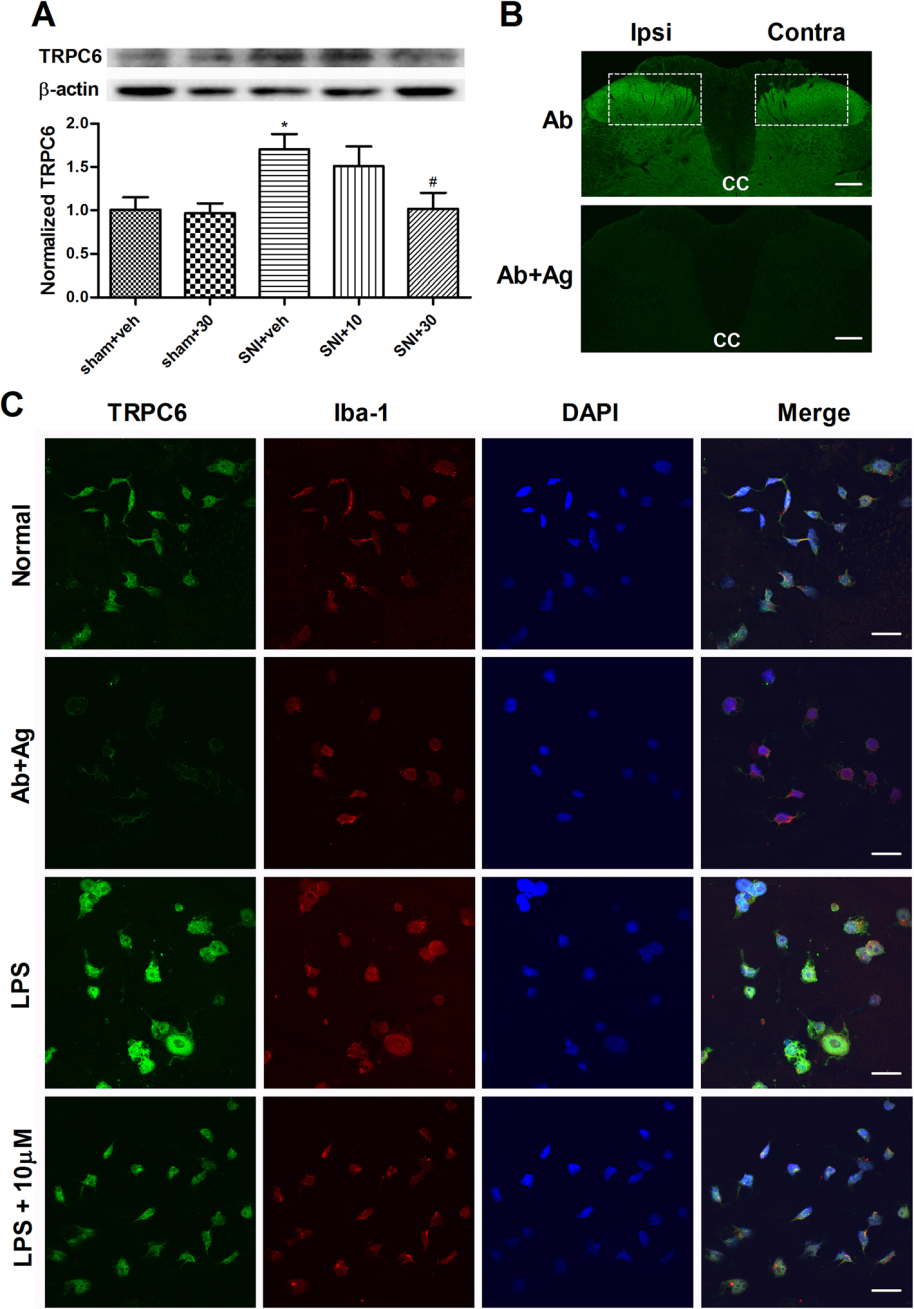
Expression feature of TRPC6 and the influence of LA on the protein. **a** Western blot analysis showed that nerve injury increased the level of TRPC6 protein in the ipsilateral SDH, which was dose dependently downregulated by LA at 10 and 30 μM. Upper panel, typical blotting band. Lower panel, summarized analysis of TRPC6 in five groups. *p < 0.05 vs. sham + veh group; ^# ^*p* <0.05 vs. SNI + veh group; *n* = 5. **b** Typical picture of TRPC6 immunostaining in the SDH of vehicle treated SNI rat. Upper panel, TRPC6 IR exhibited cloudy like even distribution in the neuropil, with preference in laminae I and II. The intensity of TRPC6 in the superficial dorsal horn of the ipsilateral side appeared stronger than that in the contralateral side, mainly within the medial section (denoted by the white rectangle). Lower panel, control picture of TRPC6 staining after the antibody was pre-absorbed by the antigen. *CC* central canal, *Ipsi* ipsilateral side, *Contra* contralateral side, *Ab* antibody, *Ag* antigen. Scale bar, 200 μm. **c** Triple-labeled staining of TRPC6, Iba-1, and DAPI in cultured microglia. First panel, normal staining showing that TRPC6 was mainly distributed in the membrane, cytosol and the processes of Iba-1-labeled microglia. Second panel, normal cells with pre-absorbed TRPC6 antibody staining. Third panel, triple staining after the cells were challenged by LPS at 500 ng/mL for 24 h. Microglia showed the typical M1 phenotype with hypertrophied cell body and short but thick processes. TRPC6 and Iba-1 IR were greatly elevated in the cells. Last panel, co-incubation of the cell with 10 μM of LA and LPS markedly decreased TRPC6 and Iba-1 IR with the regression of the cell shape. Scale bar, 30 μm

The results hitherto strongly demonstrate the role of LA in suppression of microglia activation and the increase of proinflammatory cytokines in the spinal cord. Since LA is a nominated TRPC6 inhibitor, it is intriguing to know the expression property of TRPC6 in the spinal cord and the influence of LA on this channel. As shown in Fig. 8a, Western blot assay detected a band around 100 kD from the tissue of ipsilateral SDH. Nerve injury caused a significant increase of the band density on POD 6, which was however dose-dependently downregulated by the *i.t.* application of LA at 10 and 30 μM (Fig. 8a; *p* < 0.05, *n* = 5). Immunofluorescent staining demonstrated that TRPC6 IR in the spinal cord of SNI rats exhibited a cloudy like profile with a preferent location in the superficial dorsal horn, mainly laminae I and II (Fig. 8b). Careful comparison revealed that the TRPC6 IR in the ipsilateral superficial dorsal horn were apparently stronger than those in the contralateral side, especially in the medial section (denoted in while rectangles in Fig. 8b). A vague profile of the spinal cord was detected when TRPC6 antibody was pre-absorbed with its antigen, suggesting that the TRPC6 IR were mostly specific.

Due to the low resolution of tissue staining of TRPC6 in recognizing definite cells, we further examined its expression in cultured microglial cells and tested the influence of LA after LPS challenge, with triple-labeled immunocytochemical staining. As demonstrated in Fig. 8c, TRPC6 IR were clearly detected in the perikarya area of the cytosol, plasma membrane, and the thin processes of Iba-1-labeled normal microglia. The staining was, however, only weakly detected in the cell when TRPC6 antibody was pre-absorbed with its antigen (the second panel in Fig. 8c). Incubation with LPS at 500 ng/mL for 24 h caused the obvious increase of TRPC6 IR and Iba-1 IR in microglia that showed hypertrophied cell body and short but thick processes, featured the M1 phenotype. The enhanced IR of TRPC6 and Iba-1 were effectively inhibited by co-incubation with LA at either 10 or 30 μM (data for 30 μM not shown), accompanied by the recovered shape of microglia.

These results strongly suggest that nerve injury or LPS stimulation induces the increased expression of TRPC6 in the microglia, which can be efficiently downregulated by the application of LA.

### The effects of TRPC6 knockdown

#### On neuropathic pain behaviors

**Fig. 9:**
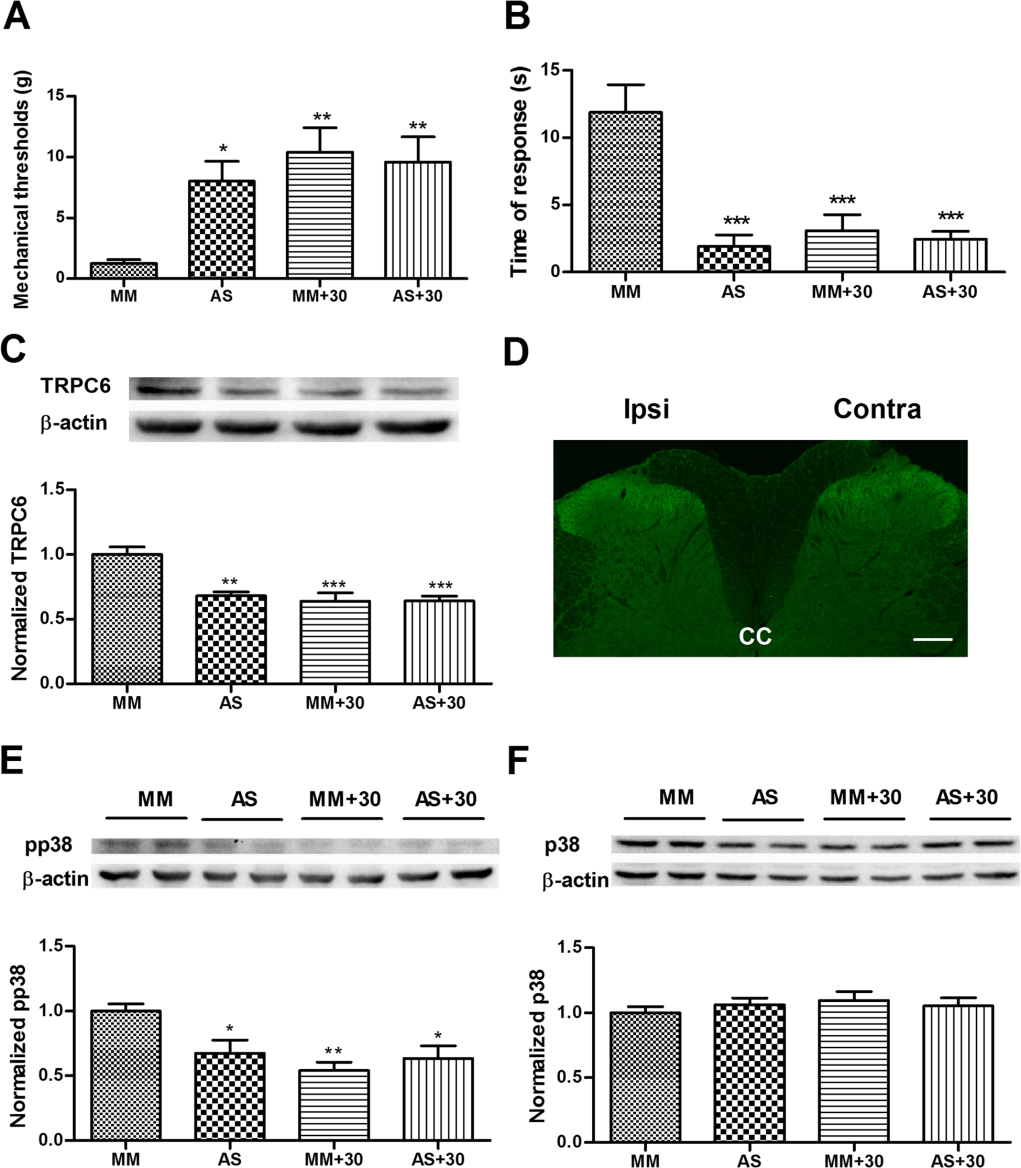
The changes of pain behaviors and p38 signaling after knockdown of TRPC6. **a** von Frey filament tests showing that *i.t.* application of TRPC6 antisense (AS) for 6 days significantly relieved mechanical hypersensitivity in SNI rats compared with mismatch (MM) treatment group. Supplementation with 30 μM of LA rescued the effect of sole mismatch while had no obvious influences on the effect of sole antisense. *n* = 6–8. **b** Acetone test showing that treatment with TRPC6 antisense also remarkably suppressed cold allodynia compared with mismatch group. This effect was almost equal to that of the co-administration groups. *n* = 6–9. **c** Western blot analysis demonstrated that application of antisense ODN caused significant decrease in the level of TRPC6, the efficacy of which was comparable to the other two groups. *n* = 5. **d** Typical picture of TRPC6 immunofluorescent staining illustrating the generally decreased intensity of TRPC6 IR after antisense treatment. *CC* central canal. Scale bar, 200 μm. **e**, **f** Western blot analysis demonstrated that phosphorylated p38 (**e**) other than p38 (**f**) was significantly suppressed in the antisense and the two co-administration groups. *p < 0.05, **p <0.01, ***p < 0.001 vs. mismatch group, *n* = 5

In addition to TRPC6, LA also exerts blocking effects on TRPC3 and TRPC7 with low selectivity [18]. To examine if the observed analgesic and anti-inflammatory effects involve other TRP isoforms and channels, rats received *i.t.* administration of antisense or mismatch ODN for TRPC6, daily for 5 days. As shown in Fig. 9a, b, TRPC6 antisense significantly inhibited the decrease in mechanical thresholds and the increase in cold response induced by nerve injury compared with mismatch treated group (*p* < 0.05, *p* < 0.001 vs. mismatch respectively; *n* = 6–8). Interestingly, co-administration of LA at 30 μM with mismatch markedly rescued the effect of sole mismatch, whereas had no further influences on the effect of sole antisense, suggesting that the efficacy of TRPC6 antisense is comparable to that of LA at 30 μM. To confirm the behavioral observation, Western blot and immunostaining were performed after the final application of ODN/drug on POD 6. As shown in Fig. 9c, TRPC6 antisense caused 32.0 ± 3.2% reduction in the expression level of TRPC6 protein compared with the mismatch group (*p* < 0.01, *n* = 5). It also caused decreased immunoreactive intensity of TRPC6 in the SDH without changing the distribution feature of the channel (compare Fig. 9d with the upper panel in Fig. 8b). The knockdown efficacy was comparable to that of the two groups with co-administration (36.2 ± 6.7% in MM + LA group; 35.9 ± 3.8% in AS + LA group). These results suggest that increased expression of TRPC6 in the spinal cord may contribute to the formation of neuropathic pain, and the analgesic effects of LA might be ascribed to the downregulation of TRPC6 therein.

#### On p38 MAPK signaling

It is well known that p38 pathway contributes to the activation of microglia in the spinal cord [25]. We questioned if downregulation of TRPC6 affected the level of this signaling. As shown in Fig. 9e, Western blot assay revealed that treatment with TRPC6 antisense significantly decreased the level of pp38 compared with mismatch group (*p* < 0.05, *n* = 5). Likewise, co-administration of LA at 30 μM with mismatch efficiently downregulated the level of pp38 compared with the sole mismatch group (*p* < 0.01, *n* = 5). Nonetheless, supplementation of antisense with LA did not cause further decrease of pp38 level compared with the effect of sole antisense, suggesting that the actions of LA and TRPC6 antisense are likely to converge on the same downstream molecule, pp38. In contrast to these changes, the level of p38 kept almot intact in the four groups (Fig. 9f). 

**Fig. 10:**
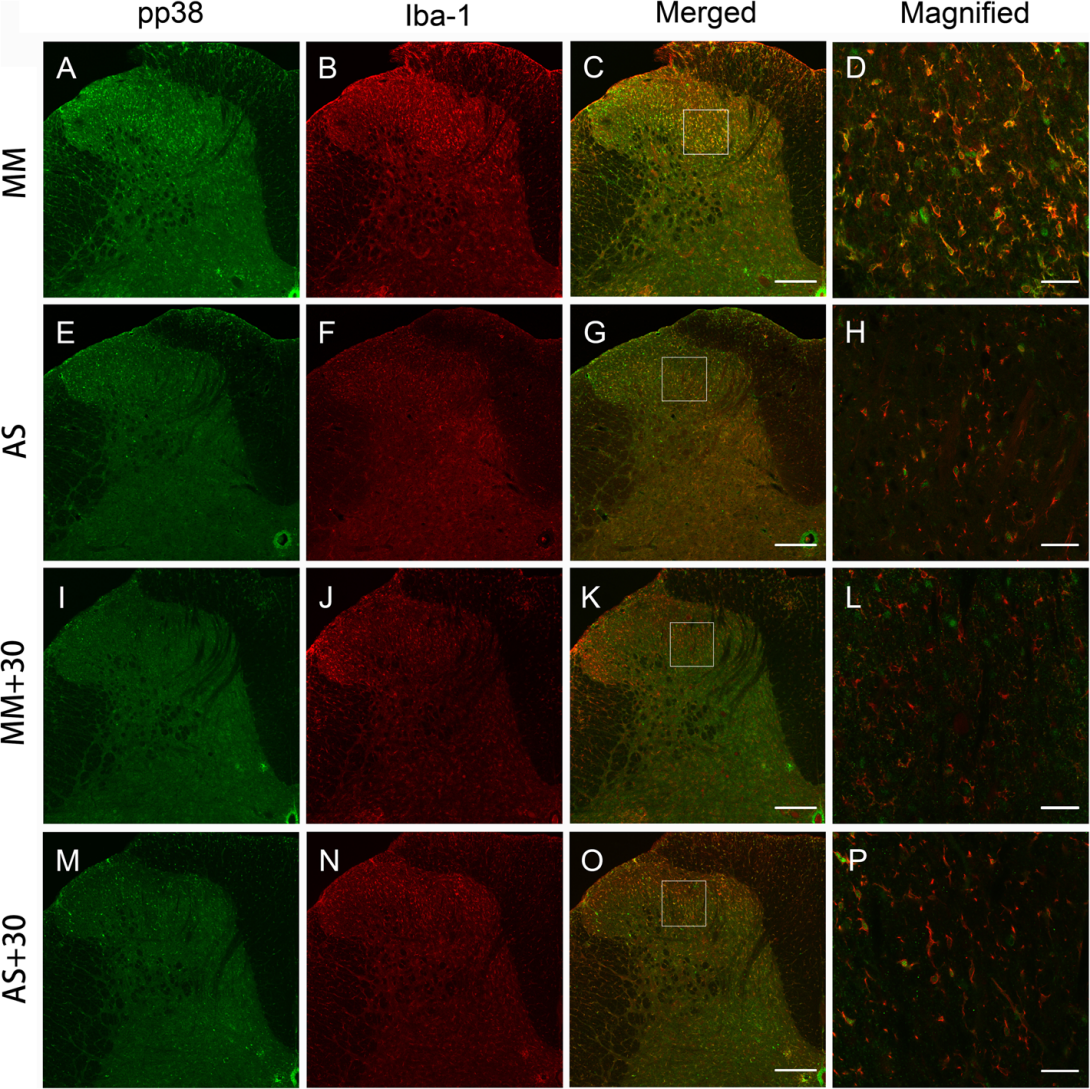
The effects of TRPC6 knockdown and the co-administration with LA on pp38 immunofluorescent staining in SNI rats. **a**–**d** Mismatch group, **e**–**h** antisense group, **i**, **j** mismatch supplemented with LA at 30 μM, **m**–**p** antisense supplemented with LA at 30 μM. pp38 IR (in green) in the mismatch group were markedly increased in the medial two thirds part of the ipsilateral SDH, compared to the sporadic distribution in the contralateral side (data not shown). Most of pp38 IR were co-existed with Iba-1, suggesting the dominant localization in the microglia (**d**). Antisense or the two co-administrations caused prominent decrease of pp38 IR and Iba-1 IR (in red). **d**, **h**, **l**, **p** Illustrated the magnified images enclosed in **c**, **g**, **k**, **o**, respectively. Nerve injury caused the upregulation of pp38 IR in the soma and processes of microglia (**d**). But those in the processes were markedly reduced in the antisense and co-administration groups (**h**, **l**, **p**). Scale bar, 30 μm in **d**, **h**, **l**, **p**; 100 μm in the remaining pictures

We finally performed double immunofluorescent staining and investigated the distribution changes of pp38 among the four groups. As shown in Fig. 10a, nerve injury in mismatch group caused remarkable increase of pp38 IR in the medial two thirds of the ipsilateral SDH compared with the contralateral side (data not shown). Most of the pp38 IR were elegantly overlapped with Iba-1-labeled microglia in both soma and processes of the cell (Fig. 10b–d), suggesting the intensive upregulation of pp38 in the microglia. Treatment with TRPC6 antisense or the two combinations caused striking decrease of pp38 IR, accompanied by the reduced detection of Iba-1 IR in the dorsal horn (Fig. 10e-p). Notably, magnified illustrations demonstrated that the decrease of pp38 IR in the processes of microglia was particular prominent, as evidenced by the almost sole Iba-1 staining therein (Fig. 10h, l, p). Together, these results suggest that the phosphorylation level of p38 in the SDH is correlated with the expression of TRPC6 in the current neuropathic pain model, and the analgesic effects of LA involve the downregulation of the spinal TRPC6 and p38 signaling.

## Discussion

The present study demonstrated that single and multiple *i.t.* treatments with LA effectively suppressed mechanical hypersensitivity and cold allodynia in the rat model of neuropathic pain induced by SNI. The analgesic action was associated with inhibition of the overactivity of microglia and the upregulation of proinflammatory cytokines such as TNF-α, IL-1β, and IL-6, whereas had no obvious corelations with the activity of astrocytes and the level of IL-10. The mechanisms responsible for the action involves the downregulation of TRPC6 channel and its downstream p38 signaling in the microglia. To our knowledge, this is the first report to show the analgesic effects of LA, a potent inhibitor of TRPC6 channel.

### The analgesic efficacy of LA in neuropathic pain

LA is a larch-derived labdane-type diterpene that exhibits TRPC6-inhibitory biologic activity [18]. It has been shown that within the range of 0.1–10 μM, LA dose-dependently inhibited Ca^2+^ entry mediated by stably expressed TRPC6 in HEK293 cells with the IC_50_ value of about 0.58 μM. This action was confirmed in primary cultures of pulmonary artery smooth muscle cells and isolated mouse lungs where TRPC6 is highly expressed, at the dose of 5 μM [18]. It is thus evident that LA at the range of submicromolar and micromolar is sufficient to suppress both heterologously expressed and native TRPC6 channel activity. Given the reasonable dilution of intrathecal applied drug, the dose range between 3 and 30 μM with the volume of 10 μL for each application was chosen to examine its effects on animal pain behaviors.

As a result, single application of LA dose-dependently ameliorated animal mechanical allodynia in SNI-induced neuropathic pain model with the ED_50_ value of 13.43 μM. This value is close to the effective dose demonstrated in native smooth muscle cells [18]. Of note, single application of LA at 30 μM caused about 70% restoration of mechanical threshold (9.33 ± 2.03 g in SNI + 30 μM vs. 13.17 ± 1.83 g in sham + vehicle) lasting for 1.5 h (from 90 min to 180 min post drug). And the efficacy of the drug could be maintained to the next day after multiple applications, suggesting the potent action of the drug in treating mechanical pain.

Cold hypersensitivity is also an essential manifestation of neuropathic pain induced by SNI [23]. It appeared as earlier as mechanical pain and lasted throughout the observation. Application of LA at 30 μM effectively relieved cold allodynia but with lower potency, which was supported by two evidences. Firstly, single administration of LA at 30 μM caused approximate 59% improvement of cold response (6.73 ± 1.25 s in SNI + 30 μM vs. 16.56 ± 2.88 s in SNI + vehicle). Secondly, its analgesic action could not be well maintained to the next day after multiple applications (compare the two “pre” columns in Fig. 3b, d). Since there is no report that TRPC6 has sensitivity to thermal stimulation, we speculate that this effect may be secondary to the improvement of peripheral and/or central sensitization by LA.

The dose of LA below 50 μM has been proved to be non-toxic to cells in both short-term and long-term incubation [18]. These results suggest that multiple *i.t.* applications of LA at 30 μM may be a novel analgesic strategy in treatment of varied modalities of neuropathic pain. Interestingly, it has recently been suggested that *i.p.* application of LA at 5 mg/kg/day for 7 days is also protective against traumatic brain injury-induced systemic endothelial dysfunction which is possibly mediated by TRPC6 [26]. Our results thus demonstrated another biological activity of LA in treating chronic pain with lower dose.

### The inhibition of microglia hyperactivity and the disturbance of proinflammatory cytokines

Microglia are the resident macrophage in the central nervous system. Injured primary sensory neurons release factors such as colony stimulating factor one 1, CCL2, CXCL1, caspase 6, neuregulin-1 that initiate microgliosis characterized by the morphological changes and proliferation of microglia [25]. Usually, microglia undergo morphological changes within 24 h, begin to proliferate within 2 to 3 days, and reach maximal levels in 4 to 7 days [27]. A multiple of receptors, kinases, enzymes, and marker proteins are upregulated following the activation of microglia [25], within which Iba-1 is generally accepted as the molecular marker to evaluate the level of hyperactivity of microglia. In the current study, a significant increase of Iba-1 expression was observed on day 6 post nerve injury, which is confined to the medial half of the dorsal horn, i.e. the central projection territory of the injured tibial and common peroneal nerve, manifesting the hyperactivity of microglia and/or microgliosis in the area. These changes are consistent with the abovementioned features of microglia reactions following neuropathic pain [27]. Consecutive applications of LA for 6 days dose-dependently suppressed the hyperactivity of microglia induced by SNI, as evidenced by the reduced level of Iba-1 in Western blot analysis (Fig. 5a, b), immunohistochemical (Fig. 5c–h), and immunocytochemical (the second series in Fig. 8c) stainings at both tissue and cellular levels. Inhibition of microglial activation directly relieves neuropathic pain. It is reported that *i.p.* injection of minocycline, a non-specific inhibitor of microglia, relieved mechanical hyperalgesia and allodynia induced by spinal nerve transection [28]. Specific chemogenetic modulation of microglia activation with Gq or Gi DREADD (designer receptor exclusively activated by a designer drug) was sufficient to either generate pain or attenuate neuropathic pain induced by chronic constriction injury [29]. Thus, inhibition of microglia activation induced by SNI may underpin the analgesic effects of LA.

Activated microglia release mediators such as TNF-α, IL-1β, IL-6, brain-derived neurotrophic factor and prostaglandin E2 to regulate synaptic plasticity and central sensitization thereby contribute to animal behavioral hypersensitivity [25]. In addition, cytokines may also reach the central nervous system through the neural transmission from the primary afferent fibers and/or the diffusion or transport through disrupted blood-spinal cord barriers following nerve injury [30, 31]. It is reported that exogenous applied cytokines like IL-1β were sufficient to induce central sensitization and microglia activation [32], suggesting the importance of cytokines in the generation of central sensitization. Actually, biological targeting on the disturbance of cytokines has become one intriguing direction for the clinical treatment of intractable pains [33]. In this study, we detected upregulated TNF-α, IL-1β, IL-6, other than IL-10, an anti-inflammatory cytokine, in the ipsilateral dorsal horn by multiplex measurements, suggesting the establishment of central neuroinflammation on POD 6 after SNI. The temporal feature and the fold change are consistent with those detected in the same pain models by others [14, 34, 35]. Application of LA for 6 days dose-dependently reduced the levels of TNF-α, IL-1β, and IL-6, whereas had no observable effects on IL-10, suggesting that LA possesses striking anti-inflammatory action, which might be partially due to the prominent inhibition of reactive microglia. Nevertheless, the influence of LA on the primary afferent fibers cannot be excluded as TRPC6 expressed wherein contributes to the sensitization of primary sensory neurons after nerve injury and injured primary neurons per se is supposed to initiate central neuroinflammation [12, 25]. Of note, the increased level of TNF-α appeared more sensitive to the application of LA, as manifested by the equal effects from treatment with 10 and 30 μM of LA (Fig. 7a). TNF-α is an essential proinflammatory cytokine. Intrathecal injection of TNF-α directly leads to mechanical allodynia, and TNF-α affects pain pathways by an orchestrated modulation of DRG neurons, spinal neurons, astrocytes, microglia, vascular endothelium, and even BBB permeability [36]. In addition, supraspinal TNF-α has been regarded as the common link between chronic pain and the comorbid depression [37]. The potent suppressing effects of LA on proinflammatory cytokines including TNF-α indicate that this drug may be also promising in treating other neuroinflammatory illness.

### The non-effects of LA on astrocytes

In neuropathic pain, the response of astrocytes usually follows that of microglia [38]. For instance, nerve injury causes increased transcription of microglia marker CD14 in a few hours, but the increase of astrocyte marker GFAP is not detected until 4 days after injury [39]. Based on these temporal features, we chose 6 days post injury to detect the influence of LA on both glial cells. Activated astrocytes release proinflammatory cytokines, chemokines, and other factors such as ATP and glutamate to facilitate pain signaling [40]. In addition, it can also produce substances and actively affect the phenotype of microglia [41]. It is generally accepted that the activation of microglia contributes to pain generation, whereas the activation of astrocytes contributes to pain maintenance [42]. Nevertheless, we neither detected the changes of GFAP and astrocytes in the ipsilateral dorsal horn on day 6 post injury nor the effects of LA on them were observed. These results are in agreement with some studies which suggest that the activation of astrocytes starts on day 7 post injury, peaks on 14–21 days, and lasts over a month [40, 43, 44]. Interestingly, a recent study showed that sphingosine-1-phosphate, a biological active lipid which plays an important role in regulating the growth, survival, and migration of many cells, induces TRPC6-mediated Ca^2+^ influx in astrocytes, which finally activates mitogen-activated protein kinase signaling followed by subsequent secretion of proinflammatory factors [15]. Thus, examining the effects of LA at later stage of neuropathic pain remains necessary to pinpoint whether this drug exerts actions on astrocytes.

### The molecular mechanisms underlying the analgesic and anti-inflammatory actions of LA

Our results point out that microglia are likely to be one of the essential response cell types for LA-mediated biological actions. The Ca^2+^ signaling is crucial for the activation of microglia [45]. The endogenous channel responsible for the elevation of Ca^2+^ in reactive microglia remains less studied [46]. Recent progresses, however, suggest that TRP channels may serve this function [47]. It has been shown that microglia express some TRP family members including TRPV1, TRPV2, TRPV4, TRPM2, TRPM4, TRPM7 [48]. Comparatively, the information regarding the role of TRPC channels in microglial activity is still limited [48]. The present results demonstrated that microglia expressed TRPC6 channel which was markedly upregulated following LPS challenge and further downregulated by the treatment with LA (first series in Fig. 8c). The changes of TRPC6 were also verified at the tissue level by Western blot and immunofluorescent staining, albeit no cellular resolution (Fig. 8a, b). Thus, TRPC6 appears to be both the important Ca^2+^ source for the activation of microglia and the molecular target of LA in the present system. The role of TRPC6 in promoting inflammation is consistent with several recent reports. In BV-2 microglia cells, it is demonstrated that TRPC6 is upregulated by Aβ peptides via NF-κB, which promotes the production of TNF-α, IL-1β, IL-6, and COX-2 and contributes to the damage of hippocampus neurons [14]. In bronchial epithelial cells, LPS induces the overexpression of TRPC6 and TRPC6 derived Ca^2+^ influx whereby promoting the activation of ERK1/2, p38, and NF-κB and the inflammatory responses [49]. In addition, TRPC6 has recently been shown to contribute to morphine-induced antinociceptive tolerance and hyperalgesia in rats, through similar mechanisms [50].

According to Urban et al., LA blocked TRPC6 channel via an extracellularly accessible binding site in a reversible and repeatable fashion [18]. It is thus puzzling why extracellular blocking of TRPC6 activity by LA results in the decreased expression of the channel per se. One possible mechanism may lie in the positive feedback regulation of the channel. It has been reported that angiotensin II enhances TRPC6 mRNA and protein levels in cultured podocytes and glomerular [51]. The action requires TRPC6-mediated Ca^2+^ entry and the activation of the Ca^2+^-dependent protein phosphatase calcineurin and its substrate nuclear factor of activated T cells (NFAT). Interestingly, this autoregulatory mechanism seems common among TRPC3/6/7 subfamily in triggering pathophysiological changes in different tissues. In murine heart, He et al. reported that ischemia/reperfusion increased the levels of TRPC3, TRPC6, and phosphorylated NFAT in areas-at-risk tissue, consistent with the above positive-feedback loop-directed transcription of TRPC genes [52]. It is therefore clear that the analgesic and anti-inflammatory functions of LA are probably mediated by blocking the Ca^2+^ entry and the subsequent genetic regulation of TRPC6 level.

To further evaluate the role of TRPC6, antisense experiments were finally performed. The results showed that downregulation of TRPC6 by 6-day treatment with its antisense ODN significantly improved animal mechanical and thermal allodynia, in parallel with the suppression of Iba-1 level in the microglia (Figs. 9a, b and 10b, f), suggesting that TRPC6 plays an important role in the faciliation of neuropathic pain and microgliosis. These results are in concert with the recent studies demonstrating the role of TRPC6 in varied chronic pain, such as neuropathic pain, cancer pain and morphine induced pain [12, 50, 53]. Thus, TRPC6 may be an important and promising molecular target for the management of chronic pain. Of note, the two groups of co-administration produced similar analgesic effects with similar downregulated efficacy on TRPC6 to that of antisense group, suggesting that the action of LA is probably mainly mediated by the downregulation of the channel.

Phosphorylation of p38 is a key event in the hyperactivity of microglia and plays essential roles in regulation of proinflammatory cytokines and generation of chronic pain [25, 54] . Here, we reported the intensive increase of pp38 level in microglia of the ipsilateral dorsal horn (Figs. 9e, f and 10), which was significantly inhibited by treatments with TRPC6 antisense or 30 μM of LA, suggesting that the phosphorylation of p38 may be downstream to the activation of TRPC6 and is also susceptible to the applied LA. Taken together, our results strongly demonstrate that the analgesic and anti-inflammatory actions of LA are, at least partially, mediated by the downregulation of TRPC6 channel and its downstream phosphorylation of p38 kinase.

## Conclusion

The findings presented here demonstrate that *i.t* application of LA, a nominated TRPC6 blocker, exerts strong analgesic actions in SNI-induced neuropathic pain, which may be ascribed to the TRPC6-pp38-dependent suppression of microglia activation and the anti-inflammatory roles in inhibiting the increased level of proinflammatory cytokines in the spinal cord. This action of LA is reminiscent of minocycline, the non-specific inhibitor of microglia, which shows strong analgesic effects in preclinical treatment of varied chronic pains [55]. Nevertheless, it is reported that minocycline causes partial inhibition of proinflammatory cytokines and cannot attenuate the existing neuropathic pain [28], whereas LA showed reversal activities at either single or multiple applications post injury. Thus, LA may be a novel analgesic possessing potent anti-inflammatory action in treatment of neuropathic pain. Future studies are needed to examine the universality of its analgesic action in other chronic pain models with varied application routes, and the detailed functioning mechanisms of LA, e.g., the gap between TRPC6 and pp38.

## Data Availability

All data generated or analyzed during this study are included in this published article.

## Abbreviations

DRG: Dorsal root ganglia
GFAP: Glial fibrillary acidic protein
Iba-1: Ionized calcium-binding adaptor molecule 1
IL: Interleukin
IR: Immunoreactivities
LA: Larixyl acetate
LPS: Lipopolysaccharide
POD: Postoperative day
SDH: Spinal dorsal horn
SNI: Spared nerve injury
TNF-α: Tumor necrosis factor-α
TRPC6: Transient receptor potential canonical 6
